# Reliability of Trapezius Muscle Hardness Measurement: A Comparison between Portable Muscle Hardness Meter and Ultrasound Strain Elastography

**DOI:** 10.3390/s20247200

**Published:** 2020-12-16

**Authors:** Tomonori Sawada, Hiroki Okawara, Daisuke Nakashima, Shuhei Iwabuchi, Morio Matsumoto, Masaya Nakamura, Takeo Nagura

**Affiliations:** 1 Department of Orthopaedic Surgery, Keio University School of Medicine, Shinjuku, 35 Shinanomachi, Shinjuku-ku, Tokyo 160-8582, Japan; tomonori-sawada@keio.jp (T.S.); hiroki.okawara@keio.jp (H.O.); shu1158@keio.jp (S.I.); morio@a5.keio.jp (M.M.); masa@keio.jp (M.N.); nagura@keio.jp (T.N.); 2 Diagnosis and Treatment Division, Nagura Orthopedic Clinic, Nihombashi Nomura Building (YUITO 7F) 2-4-3 Nihombashi Muromachi, Chuo-ku, Tokyo 103-0022, Japan; 3 Department of Clinical Biomechanics, Keio University School of Medicine, Shinjuku, 35 Shinanomachi, Shinjuku-ku, Tokyo 160-8582, Japan

**Keywords:** muscle hardness, trapezius muscle, muscle hardness meter, ultrasound strain elastography, strain ratio, intra-tester reliability, ergonomics

## Abstract

Prolonged computer work and smartphone use can cause stiffness of the neck and shoulder muscles, including the trapezius muscle. Hence, muscle hardness quantification is clinically beneficial. The present study aimed to examine the reliability of trapezius muscle hardness measurement using a portable muscle hardness meter and ultrasound strain elastography. Overall, 20 healthy young men participated in this study. Prior to measurement, the participant’s subjective symptoms, particularly shoulder muscle stiffness, were rated using an 11-point verbal scale. Furthermore, hardness of the right and left upper trapezius muscles was assessed. In the strain elastography assessment, muscle hardness was evaluated using strain ratio. Results showed that, in quantifying upper trapezius muscle hardness, both portable muscle hardness meter and strain elastography had an excellent intra-tester reliability (>0.9). However, the correlation coefficients between muscle hardness values assessed using a muscle hardness meter and those evaluated with strain elastography did not significantly differ, and the scores for subjective shoulder stiffness did not correspond to muscle hardness values. Therefore, the hardness of the trapezius muscle does not directly reflect the subjective shoulder stiffness. Future studies should thoroughly examine the location of the shoulder stiffness, and check whether it is accompanied by local pain or tenderness.

## 1. Introduction

In the recent years, neck and shoulder pain has become a common symptom due to prolonged intensive computer work [1,2]. In Japan, according to the Comprehensive Survey of Living Conditions 2019 of the Ministry of Health, Labor, and Welfare, shoulder stiffness is the second most common complaint (57.2 per 1000 population) after back pain among men and is the most frequent condition (113.8 per 1000 population) among women [3]. Previous studies have shown that individuals with neck or shoulder pain have stiff trapezius muscles [4,5]. Further, those with muscle stiffness are more likely to experience neck or shoulder pain [6,7]. Therefore, trapezius muscle hardness should be quantified to obtain clinically useful information and assess the effect of therapeutic interventions.

In a clinical setting, palpation is commonly and fundamentally used in evaluating resistance or deformation caused by applying an external force to tissues. Thus, studies on muscle hardness using a push-in muscle hardness meter have been conducted for a quarter of a century [8,9,10]. The muscle is generally considered “hard” when it is fatigued and “soft” when relaxed [9,10]. Thus, fatigue caused by exercise and muscle relaxation due to various treatments can be evaluated. Using a muscle hardness meter is advantageous as it is easy to carry and the technique can be learned. However, it also has some disadvantage: it can evaluate overall resistance to pressure, but not hardness specific to each tissue. That is, the superficial muscle and deep muscle cannot be separately evaluated. Then, ultrasound elastography, a technique that is more effective in noninvasively and quantitatively evaluating tissue hardness, was developed. It can easily show the distribution of hardness within a target tissue in real time. Moreover, in recent years, it has been used in evaluating muscle hardness. In particular, shear wave elastography [11,12,13,14,15,16,17] and strain elastography [11,18,19,20,21,22,23] are commonly utilized to evaluate muscle hardness. They can identify the distribution of stiffness or hardness within a target muscle. Inami et al. [23] mentioned the characteristics of the two methods. Shear wave elastography can evaluate muscle hardness along the muscle shortening direction, and strain elastography can assess strain representing muscle hardness in a perpendicular direction to muscle shortening. Akagi and Kusama [24] evaluated trapezius muscle hardness using shear wave elastography and a muscle hardness meter. Results showed no correlation between the obtained values. Muscle hardness value obtained using strain elastography is more similar to that measured with a muscle hardness meter than that assessed using shear wave elastography. However, previous studies have not assessed the reliability and reproducibility of strain elastography and muscle hardness meter in evaluating trapezius muscle hardness.

Therefore, the present study aimed to examine the reliability of trapezius muscle hardness measurement using a muscle hardness meter and ultrasound strain elastography. We hypothesized that both methods can have good to excellent reliability and that muscle hardness value assessed using a muscle hardness meter will correspond to that obtained using strain elastography to some extent. Moreover, the correlation between the values obtained using these methods and subjective shoulder fatigue levels was investigated.

## 2. Materials and Methods

### 2.1. Participants

Published recommendations of sample size requirements vary, but it has been suggested that minimum 20 participants are sufficient for reliability studies [25]. In addition, previous studies using ultrasound strain elastography have enrolled <20 participants [18,19,20,21,23]; therefore, n = 20 was selected for the present study. Asymptomatic volunteers with no orthopedic abnormalities of the neck and shoulders aged >16 years were recruited from one hospital and one university. Therefore, 20 healthy young male individuals were included in this study. Sociodemographic characteristics (age, height, weight) were recorded. Participants were also asked about their dominant hand, smartphone use, and typing per day (Table 1). Moreover, the purpose of this study was explained to all participants, and informed consent was obtained prior to their participation. The experimental design was approved by the P-One Clinic Ethical Committee and the Institutional Review Board of Keio University School of Medicine (approval number: 20190326). This study was conducted in accordance with the Declaration of Helsinki.

### 2.2. Experimental Protocol

**Figure 1:**
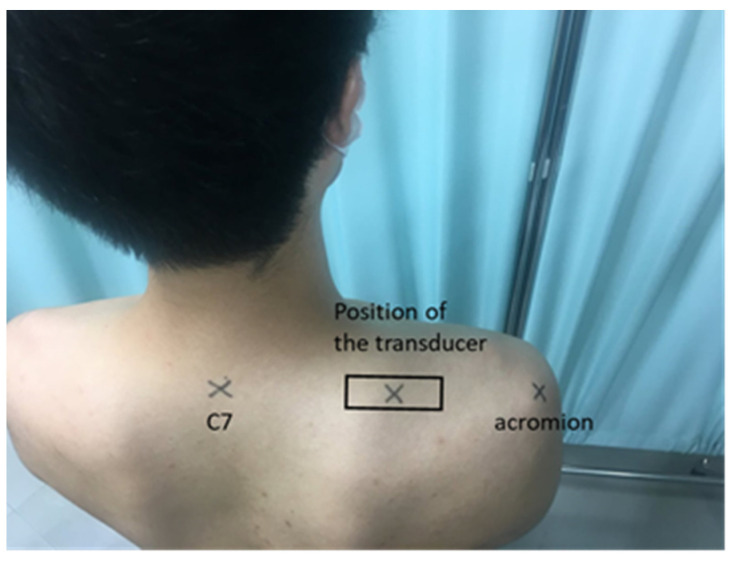
The assessment site of the upper trapezius muscle was defined as the midpoint between the C7 spinous process and acromion.

Prior to the assessment, the participants rated their current subjective symptoms, particularly shoulder muscle stiffness, using an 11-point verbal scale (0, no symptoms; 10, intolerable symptoms). Subsequently, right and left upper trapezius muscle hardness was assessed. The middle point between the seventh cervical spinous process and the tip of the acromion was selected as the measurement point (Figure 1). The right and left trapezius muscles of each participant were assessed using a muscle hardness meter and ultrasound strain elastography on the same day, and the order of measurements was random. During the evaluation, the participants sat in a height-adjustable chair with low back support, knees and hips bent at a 90° angle, and feet resting on the floor. Then, they were instructed to choose a comfortable sitting posture while maintaining the line of sight at eye level and resting their hands on the thighs with palms facing down [26].

### 2.3. Assessments

#### 2.3.1. Muscle Hardness Meter

Muscle hardness was quantitatively evaluated using a portable muscle hardness meter (NEUTONE TDM-Z2; TRY-ALL Corp., Chiba, Japan). A similar device has been used in previous studies [24,27]. There is a spring in the part that should be grasped above the display, and the hemispherical indenter at the bottom end was pushed back into the body when the indenter comes in contact with an object. Thus, the reaction force that the indenter receives from the object when a pushing force reaches approximately 14.71 N (1.5 kgf) could be assessed. The values measured using this device have no units (displayed between 0 and 100). Hence, based on the manufacturer‘s report, they were converted to Newton using the following formula: N = 0.0238 × measured value + 0.532. This was performed five times at each measurement time, and the mean value of the five trials was used as the muscle hardness value.

#### 2.3.2. Ultrasound Strain Elastography

**Figure 2:**
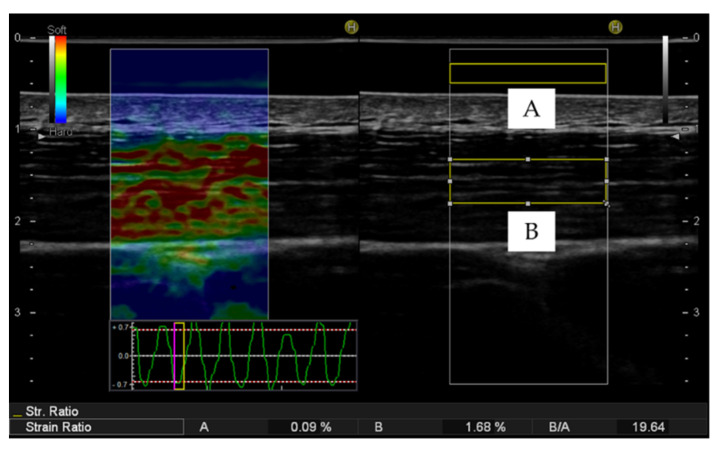
Example of strain elastography image and strain ratio. The region of interest (ROI) was set for the acoustic coupler (**A**) and on an area of the trapezius muscle (**B**). Strain ratio was defined as B/A. In the strain elastography image, the red color represents a soft area; blue, hard area; and green, an area in between soft and hard. The strain graph depicts the average tissue strains in response to pressure force. The ROI was set for the acoustic coupler (**A**) and on an area of the trapezius muscle (**B**). Strain ratio was defined as B/A. In the strain elastography image, the red color represents a soft area; blue, hard area; and green, an area in between hard and soft. The strain graph depicts the average tissue strains in response to pressure force.

Muscle strain was assessed using a diagnostic ultrasound system (HI VISION Preirus; Hitachi, Ltd., Tokyo, Japan) with a linear array transducer (EUP-L75; Hitachi, Ltd., Tokyo, Japan). An acoustic coupler (EZU-TECPL1; Hitachi, Ltd., Tokyo, Japan), with an elastic modulus of 22.6 ± 2.2 kPa, was attached to the head of the transducer with a plastic attachment (EZU-TEATC2; Hitachi, Ltd., Tokyo, Japan). During strain elastography, the investigator manually and rhythmically compressed the transducer against the participant’s trapezius muscle surface by checking the strain graph displayed on the screen of the ultrasound system (Figure 2). The compression frequency was adjusted to approximately 3 Hz. Then, color-coded images from red (soft) to blue (hard), representing the relative strain under the transducer, were generated. To convert qualitative data to semiquantitative data, two regions of interest (ROIs) for strain measurement were set for the acoustic coupler and on an area of the trapezius muscle in each elastography image. The ROIs were placed on approximately the middle third of the depth of the trapezius muscle and the acoustic coupler to prevent the influence of borderline effects, according to previous studies [21,22]. In addition, muscle hardness was evaluated using strain ratio, defined as the ratio of strain in the trapezius muscle to that in the acoustic coupler [21,22,23]. Thus, a higher strain ratio indicates a softer muscle, and a lower strain ratio indicates a harder muscle. Based on these findings, five images were randomly selected based on a clear color presence of muscle tissue and the acoustic coupler and a constant pressing force between −0.7 and 0.7 points on the strain graph during compression and release cycles [23]. Then, the average strain ratio of the five images was used as the muscle elasticity value.

### 2.4. Statistical Analysis

The intraclass correlation coefficients (ICCs) for the five repeated measurements at each assessment time were calculated to evaluate the intra-tester reliability of the muscle hardness value and the strain ratio (95% lower and upper confidence intervals). In order to investigate the relationship between muscle hardness values obtained with a muscle hardness meter and those evaluated using strain ratio, the Spearman’s rank correlation coefficient (*rs*) was calculated at each assessment site. In addition, the relationship between these muscle values, excluding outliers which is defined as values of >1.5 times than the interquartile range, was also evaluated. Furthermore, the relationship between muscle hardness values and the scores for subjective shoulder stiffness was assessed with the Spearman’s rank correlation coefficient. Data were analyzed using the Statistical Package for the Social Sciences software version 25.0 (IBM Corp., Armonk, NY, USA), and a *p* value of <0.05 was considered statistically significant.

## 3. Results

**Figure 3:**
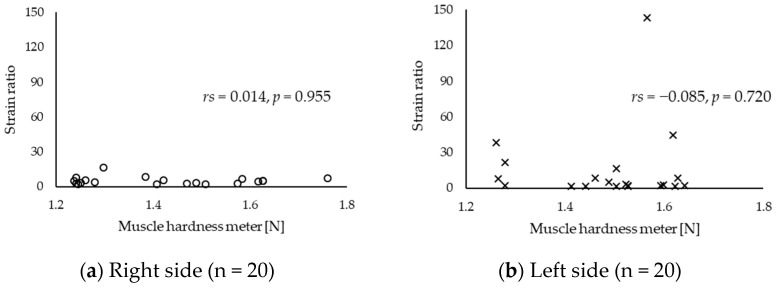
Scatter diagram between muscle hardness value assessed with a muscle hardness meter and that evaluated with strain ratio: (**a**) right side and (**b**) left side.

**Figure 4:**
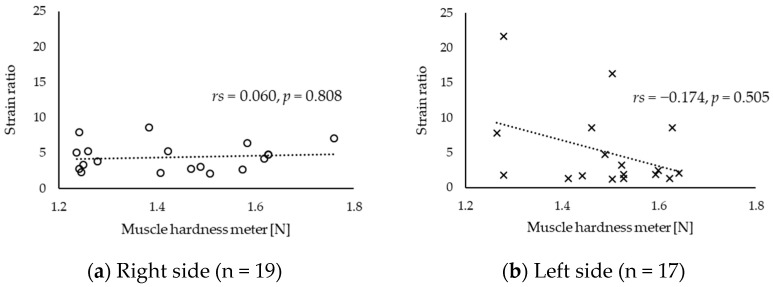
Scatter diagram between muscle hardness value assessed with a muscle hardness meter and that evaluated with strain ratio without outliers: (**a**) right side and (**b**) left side.

Table 2 depicts the intra-tester reliability of muscle hardness obtained using a muscle hardness meter and strain ratio. The ICCs of values obtained using a muscle hardness meter and strain ratio revealed an excellent reliability (>0.9). Table 3 shows the upper trapezius hardness value. Since these values did not have a normal distribution, they were presented as median and interquartile range. Based on the correlation analysis, the correlation coefficients between the muscle hardness values assessed with a muscle hardness meter and those evaluated with strain ratio did not significantly differ (Figure 3, right side: *rs* = 0.014, *p* = 0.955; left side: *rs* = −0.085, *p* = 0.720). In addition, although outliers were also considered, no statistically significant correlation was found (Figure 4).

**Figure 5:**
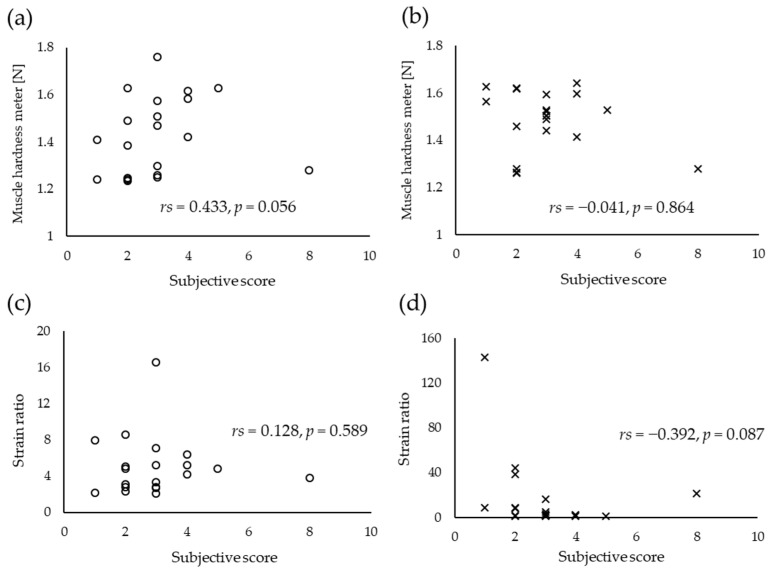
Relationship between subjective and objective shoulder stiffness (n = 20). Muscle hardness meter for the (**a**) right and (**b**) left sides. Strain ratio for the (**c**) right and (**d**) left sides evaluated using strain elastography.

Then, the relationship between shoulder complaints as subjective parameters and muscle hardness measures as objective parameters was investigated. However, the correlation coefficients between the scores for subjective shoulder stiffness and muscle hardness value for both sides did not significantly differ based on two measurements (Figure 5). Thus, the cause of subjective symptoms was not muscle hardness.

## 4. Discussion

This study examined the reliability of trapezius muscle hardness value assessed using a portable muscle hardness meter and ultrasound strain elastography. Muscle hardness was defined as resistance or deformation of the muscle to vertical pressure from above the skin. Results showed that both assessment methods had a high intra-tester reliability, and there was no correlation between muscle hardness values assessed using a muscle hardness meter and those evaluated with strain elastography. This result was contrary to our hypothesis that muscle hardness assessed using a muscle hardness meter corresponds to that investigated with strain elastography. The hardness measurement depends highly on the size of the contact area and the material of the impactor to some extent. The muscle hardness meter has a 15-mm radius impactor that is made of aluminum alloys, whereas the strain elastography is made of elastomer resin with a 600-mm^2^ (12 × 50 mm) contact area to the measured area. The difference in the two measurement systems may explain the absence of correlation in the results.

According to previous studies, the ICCs of upper trapezius muscle hardness values obtained using a muscle hardness meter and strain elastography were 0.955–0.986 (1, 5) [24] and 0.994 (1, 3) [22], respectively. Thus, the present study confirmed a similar level of reliability. Compared with shear wave elastography, strain elastography is similar to a push-in muscle hardness meter or palpation for the assessment of muscle hardness using slight manual compression in a vertical direction to the tissue. Ariji et al. [27] assessed masseter muscle hardness using a muscle hardness meter and strain elastography. Results showed that there was a significant correlation between the values obtained with a muscle hardness meter and those assessed using strain ratio. Contrarily, this study showed no correlation between these values. In terms of trapezius muscle hardness, Akagi and Kusama [24] showed that there was no association between muscle hardness meter and shear wave elastography values. Based on the abovementioned results, the insufficient relationship between these values may be more likely influenced by differences in targeted body parts rather than variations in elastography techniques. Muscle hardness meter is measured from the surface of the body according to pressure and is constantly influenced by the skin, subcutaneous adipose tissue, muscle located below the target muscle, and bone [8,10]. Therefore, the trapezius muscle itself may not be sufficiently deformed due to the thickness of the subcutaneous adipose tissue or the hardness of the deep muscle (such as the levator scapulae) that may add to the relatively large reaction force applied to the indenter of the device. Our findings showed that muscle hardness meter had high reliability, and it could be an objective index of overall tissue hardness when pressure is applied on the body surface. However, it did not solely reflect trapezius muscle tissue hardness.

This study also investigated the relationship between shoulder complaints as subjective parameters and muscle hardness as objective parameters. Results showed that the correlation coefficients between the scores for subjective shoulder stiffness and muscle hardness values on both sides did not significantly differ based on two measurements. This result was consistent with that of the study of Akagi and Kusama [24]. Contrarily, previous studies have reported that individuals with nonspecific neck/shoulder pain have a higher muscle hardness [22,28,29]. In these studies, the inclusion criteria included patients with persistent symptoms, including pain. Our study found that subjective shoulder stiffness without pain did not indicate a significant increase in muscle hardness among young male individuals. There were wide variations in neck muscle morphology among the patients with chronic neck pain [30], and it remains unclear whether we were able to identify the morphological change in the patients only by superficial measurements such as muscle hardness. Myofascial trigger points (MTrPs) have been defined as a highly localized hyperirritable spots in a palpable taut band of skeletal muscle that are spontaneously painful [31] and are thought to be caused or exacerbated by trauma, overuse, mechanical overload, postural faults or psychological stress [32]. The upper trapezius muscle has been reported to be often affected by MTrPs [33,34,35]. However, Andersen et al. [36] reported that severe tenderness occurs more commonly in the levator scapulae, neck extensors, and infraspinatus than in the trapezius muscle among generally healthy adults with nonspecific neck/shoulder pain. Hence, future studies should be conducted to evaluate in detail where shoulder stiffness occurs and whether it is accompanied by pain or MTrPs in that area. These practices will increase the usefulness of the objective assessment of muscle hardness using the muscle hardness meter and ultrasound strain elastography.

The present study has several limitations. First, in the measurement using ultrasound strain elastography, manual compression is used to produce strain in the target muscle. Therefore, control among examiners regarding the technique of compression is difficult. Second, although previous studies have shown changes in trapezius muscle hardness after static or dynamic exercise [37] and computer work [38] among healthy participants, lifestyle habits, which can affect muscle hardness, were not controlled before each assessment in this research. Recent developments in wearable sensors have made it possible to record sedentary activity time and provide feedback on poor sitting posture in daily life [39,40], and their use may improve this problem. Third, because this study included healthy college-age (range, 19–22 years) male individuals, the relationship between pain and muscle hardness could not be validated. Fourth, chronic nonspecific neck/shoulder pain was found to favor the levator scapulae, infraspinatus, and neck extensor such as the multifidi and splenius cervicis muscles [4,36]. However, we assessed only the trapezius muscle, which is the primarily affected neck and shoulder muscle. Thus, the presence of pain or MTrPs in these muscles could have caused subjective shoulder stiffness. Further studies should be conducted to investigate whether the hardness in these muscles differs between individuals with and without neck and shoulder pain.

## 5. Conclusions

The portable muscle hardness meter and ultrasound strain elastography had excellent intra-tester reliability in quantifying trapezius muscle hardness. However, there was no correlation between the muscle hardness values obtained using a muscle hardness meter and those assessed using strain elastography. Hence, these values might differ. Further, the scores for subjective shoulder stiffness did not correspond to muscle hardness values. Our results suggest that trapezius muscle hardness does not directly reflect the subjective shoulder stiffness, and future studies are needed to evaluate in detail where shoulder stiffness occurs and whether it is accompanied by pain or tenderness in that area.

## Figures and Tables

**Table 1:** Participant characteristics (n = 20).

Age (years)	20.3 ± 0.6
Height (m)	1.74 ± 0.1
Weight (kg)	71.0 ± 12.2
BMI (kg/m^2^)	23.4 ± 3.0
Dominant hand (n)	Right, 19; left, 1
Duration of smartphone use per day (hours)	5.4 ± 2.7
Duration of typing per day (hours)	1.8 ± 1.6

**Table 2:** Intra-tester reliability of upper trapezius hardness value.

	Single Measurements		Average Measurements	
	ICC (1, 1)	95% CI	ICC (1, 5)	95% CI
Right muscle hardness meter (N)	0.974	0.951−0.988	0.995	0.990−0.998
Left muscle hardness meter (N)	0.962	0.929−0.983	0.992	0.985−0.996
Right strain ratio obtained using strain elastography	0.942	0.893−0.974	0.988	0.977−0.995
Left strain ratio obtained using strain elastography	0.930	0.874−0.968	0.985	0.972−0.993

**Table 3:** Data on upper trapezius hardness value (n = 20).

	Muscle Hardness Meter (N)	Strain Ratio
Right side	1.43 (1.26, 1.58)	4.50 (2.79, 5.53)
Left side	1.51 (1.43, 1.59)	2.80 (1.79, 10.5)

Values were presented as median (first, third quartiles).
